# [1,2-Bis(diiso­propyl­phosphan­yl)ethane-κ^2^ *P*,*P*′](2-fluoro-*N*-{[(2-fluoro­phen­yl)aza­nid­yl]carbon­yl}anilinido-κ^2^ *N*,*N*′)nickel(II)

**DOI:** 10.1107/S2414314620006495

**Published:** 2020-05-19

**Authors:** Marcos Flores-Alamo, Francisco J. Perez-Ortiz, Alma Arevalo, Juventino J. Garcia

**Affiliations:** a Facultad de Química, Universidad Nacional Autónoma de México, Ciudad, Universitaria, Ciudad de México, 04510, Mexico; Vienna University of Technology, Austria

**Keywords:** [1,2-bis­(diiso­propyl­phosphino)ethane]­nickel(II), bis­(2-fluoro­phen­yl)urea, square-planar coordination environment, crystal structure

## Abstract

The mol­ecular structure of the title complex, [Ni(C_13_H_8_F_2_N_2_O)(C_14_H_32_P_2_)] or Ni(oFPU)(dippe), where oFPU is the dianion of bis­(2-fluoro­phen­yl)urea and dippe is 1,2-bis­(di-iso­propyl­phosphino)ethane, comprises an Ni^II^ atom with a distorted square-planar coordination environment (geometry index τ_4_ = 0.195). One of the fluoro­phenyl rings of the oFPU ligand is disordered over two sets of sites in an 0.832 (7):0.168 (7) ratio. The crystal structure displays C—H⋯O and C—H⋯F hydrogen-bonding inter­actions, leading to chains with *R*_2_^2^(12) motifs extending parallel to [100]. The title compound might be of inter­est with respect to the production of urea and carbamate derivatives of nickel(II).

## Abstract 2

The Ni^II^ atom in the title compound shows a distorted square-planar P_2_N_2_ coordination set provided by two bidentate ligands.

## Graphical abstract

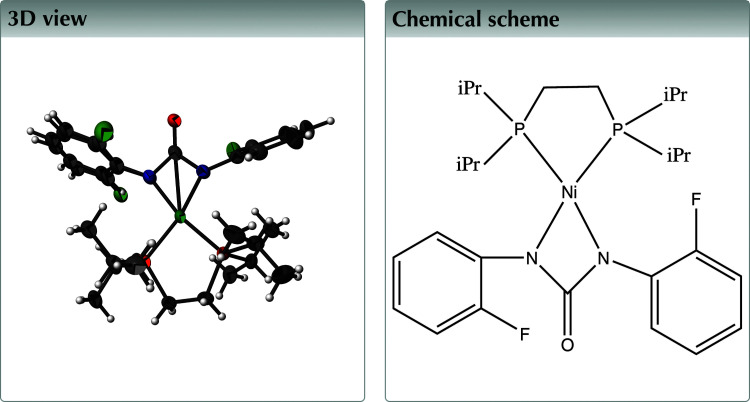

## Structure description

Iso­cyanates are compounds that contain the functional group –N=C=O and can be prepared in different ways, from rearrangements on a laboratory scale to the phospho­genation of primary amines (Saunders & Slocombe, 1948 ▸) used in industry. The importance of iso­cyanates is demonstrated by the multitude of reactions in which they can be either used directly or serve as reaction inter­mediates. For example, iso­cyanates are employed in the production of urea and carbamate derivatives, which find agrochemical and/or pharmaceutical applications (Braunstein & Nobel, 1989 ▸). In this context, studies regarding the reactivity of aromatic iso­cyanates with different substituents on the aromatic rings in the dimeric complex [Ni(dippe)]_2_(*μ*-H)_2_ [dippe = 1,2-bis­(diiso­propyl­phosphino)ethane], which is an excellent precursor of nickel(II), were started.

**Figure 1:**
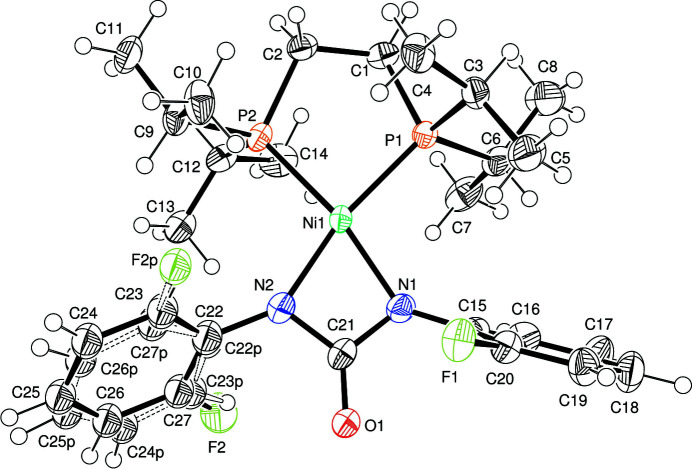
The mol­ecular structure of [Ni(oFPU)(dippe)], showing the atom labelling. Displacement ellipsoids are drawn at the 50% probability level.

The asymmetric unit of the title compound (Fig. 1 ▸) consists of one [Ni(oFPU)(dippe)] mol­ecule with oFPU = bis­(2-fluoro­phen­yl)urea. Both bidentate oFPU and dippe ligands are coordinated to the Ni^II^ ion, through the N and P atoms, respectively. The resulting coordination environment is distorted square-planar (Table 1 ▸), with the geometry index *τ*_4_ = 0.195 (*τ*_4_ = 0 for an ideal square-planar arrangement; Yang *et al.*, 2007 ▸). In the oFPU moiety, the fluoro­phenyl ring (F2, C22–C27) attached to N2 is disordered over two sets of sites. The aromatic rings are inclined to the NCON plane of urea by 62.90 (2) (C22–C27) and 70.58 (2)° (C15–C2); the angle between the two aromatic rings is 57.47 (2)°. Based on the relative orientation of the *ortho* substituents (considering only the major disorder component) with respect to the carbonyl group, the mol­ecular conformation can be described as *anti–anti*, showing torsion angles (O)=C21—N1—C15—C16 and (O)=C21—N2—C22—C23 of 120.9 (6) and 117.0 (5)°, respectively. These values are consistent with those reported in the literature (Solomos *et al.*, 2017 ▸).

**Figure 2:**
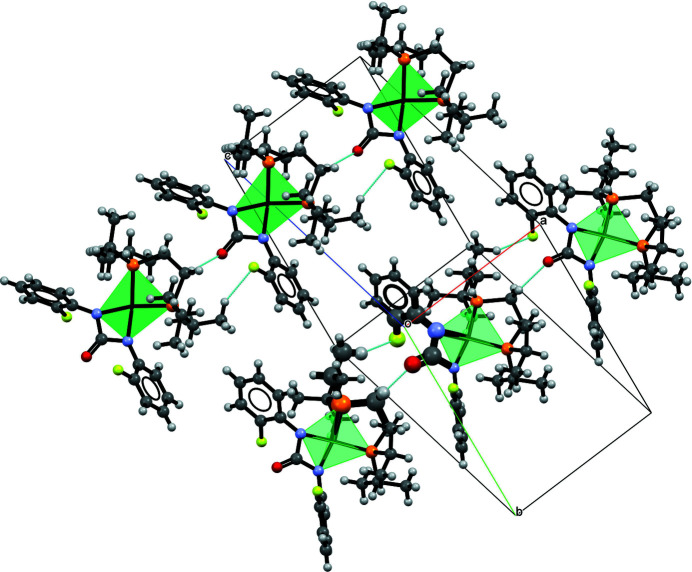
Crystal packing of [Ni(oFPU)(dippe)], showing the strongest C—H⋯O and C—H⋯F contacts as dashed lines. For clarity, only the major component of the disordered fluoro­phenyl ring (F2/C22–C27) is shown.

In the crystal packing (Fig. 2 ▸), there are inter­molecular hydrogen-bonding inter­actions between the C donor atoms of dppe to O and F acceptor atoms oFPU (Table 2 ▸). The strongest inter­actions involving C1⋯O1^i^ [3.210 (6) Å] and C8⋯F1^i^ [3.398 (8) Å] lead to the formation of chains with an 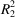 (12) motif extending along [100].

## Synthesis and crystallization

A solution of 2-fluoro­phenyl­iso­cyanate (16 mg, 0.13 mmol) in THF (5 ml) was added dropwise to a stirring THF solution of [Ni(dippe)(*μ*-H)]_2_ (35.9 mg, 0.058 mmol). A slight bubbling was observed, accompanied by colour changes from purple to green and then brown. The reaction mixture was subsequently heated at 353 K for 2 h. At the end of heating, the sample was placed in a vial, and left in an inert atmosphere for crystallization by evaporation of the solvent. After a few days, crystals formed, which were analyzed by single-crystal X-ray diffraction.

## Refinement

Crystal data, data collection and structure refinement details are summarized in Table 3 ▸. One of the fluoro­phenyl rings (F2/C22–C27) was found to be disordered over two sets of sites in a refined 0.832 (7):0.168 (7) ratio. Restraints on bond lengths, angles and displacement ellipsoids were used to model the disorder.

## Supplementary Material

Crystal structure: contains datablock(s) global, I. DOI: 10.1107/S2414314620006495/wm4130sup1.cif

CCDC reference: 2004016

Additional supporting information:  crystallographic information; 3D view; checkCIF report

## Figures and Tables

**Table 1:** Selected geometric parameters (Å, °)

Ni1—N1	1.929 (4)	Ni1—P2	2.1631 (14)
Ni1—N2	1.944 (5)	Ni1—P1	2.1729 (14)
			
N1—Ni1—N2	68.13 (19)	N1—Ni1—P1	101.11 (14)
N2—Ni1—P2	103.56 (13)	P2—Ni1—P1	87.95 (5)

**Table 2:** Hydrogen-bond geometry (Å, °)

*D*—H⋯*A*	*D*—H	H⋯*A*	*D*⋯*A*	*D*—H⋯*A*
C1—H1*B*⋯O1^i^	0.99	2.32	3.210 (6)	149
C10—H10*C*⋯F1^ii^	0.98	2.57	3.416 (7)	145
C5—H5*A*⋯F1	0.98	2.53	3.435 (8)	154
C8—H8*A*⋯F1^i^	0.98	2.56	3.398 (8)	143
C4—H4*B*⋯F2*P* ^ii^	0.98	2.5	3.25 (2)	133
C10—H10*A*⋯F2*P*	0.98	2.31	2.84 (2)	114

Symmetry codes: (i) 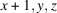 ; (ii) 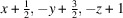 .

**Table 3:** Experimental details

Crystal data	
Chemical formula	[Ni(C_12_H_8_F_2_N_2_O)(C_15_H_32_P_2_)]
*M*_r_	567.26
Crystal system, space group	Orthorhombic, *P*2_1_2_1_2_1_
Temperature (K)	130
*a*, *b*, *c* (Å)	9.0690 (3), 14.8325 (4), 20.1784 (7)
*V* (Å^3^)	2714.32 (15)
*Z*	4
Radiation type	Cu *K*α
μ (mm^−1^)	2.45
Crystal size (mm)	0.52 × 0.43 × 0.10

Data collection	
Diffractometer	Agilent Xcalibur, Atlas, Gemini
Absorption correction	Analytical (*CrysAlis RED*; Agilent, 2013 ▸)
*T*_min_, *T*_max_	0.395, 0.79
No. of measured, independent and observed [*I* > 2σ(*I*)] reflections	29885, 5357, 5015
*R*_int_	0.069
(sin θ/λ)_max_ (Å^−1^)	0.622

Refinement	
*R*[*F* ^2^ > 2σ(*F* ^2^)], *wR*(*F* ^2^), *S*	0.048, 0.125, 1.03
No. of reflections	5357
No. of parameters	292
No. of restraints	84
H-atom treatment	H-atom parameters constrained
Δρ_max_, Δρ_min_ (e Å^−3^)	0.70, −0.57
Absolute structure	Flack *x* determined using 2039 quotients [(*I* ^+^)−(*I* ^−^)]/[(*I* ^+^)+(*I* ^−^)] (Parsons *et al.*, 2013 ▸)
Absolute structure parameter	−0.004 (14)

Computer programs: *CrysAlis PRO* (Agilent, 2013 ▸), *SHELXT2018* (Sheldrick, 2015*a* ▸), *SHELXL2018* (Sheldrick, 2015*b* ▸), *ORTEP-3 for Windows* and *WinGX* (Farrugia, 2012 ▸) and *Mercury* (Macrae *et al.*, 2020 ▸).

## full crystallographic data

### Crystal data

**Table:**

[Ni(C_12_H_8_F_2_N_2_O)(C_15_H_32_P_2_)]	*F*(000) = 1200
*M**_r_* = 567.26	*D*_x_ = 1.388 Mg m^−^^3^
Orthorhombic, *P*2_1_2_1_2_1_	Cu *K*α radiation, λ = 1.54184 Å
Hall symbol: P 2ac 2ab	Cell parameters from 12885 reflections
*a* = 9.0690 (3) Å	θ = 4.3–72.6°
*b* = 14.8325 (4) Å	µ = 2.45 mm^−^^1^
*c* = 20.1784 (7) Å	*T* = 130 K
*V* = 2714.32 (15) Å^3^	Plate, black
*Z* = 4	0.52 × 0.43 × 0.10 mm

### Data collection

**Table:**

Agilent Xcalibur, Atlas, Gemini diffractometer	5357 independent reflections
Graphite monochromator	5015 reflections with *I* > 2σ(*I*)
Detector resolution: 10.4685 pixels mm^-1^	*R*_int_ = 0.069
ω scans	θ_max_ = 73.6°, θ_min_ = 3.7°
Absorption correction: analytical (*CrysAlis RED*; Agilent, 2013)	*h* = −11→11
*T*_min_ = 0.395, *T*_max_ = 0.79	*k* = −18→18
29885 measured reflections	*l* = −24→20

### Refinement

**Table:**

Refinement on *F*^2^	Hydrogen site location: inferred from neighbouring sites
Least-squares matrix: full	H-atom parameters constrained
*R*[*F*^2^ > 2σ(*F*^2^)] = 0.048	*w* = 1/[σ^2^(*F*_o_^2^) + (0.0619*P*)^2^ + 3.6612*P*] where *P* = (*F*_o_^2^ + 2*F*_c_^2^)/3
*wR*(*F*^2^) = 0.125	(Δ/σ)_max_ < 0.001
*S* = 1.03	Δρ_max_ = 0.70 e Å^−^^3^
5357 reflections	Δρ_min_ = −0.57 e Å^−^^3^
292 parameters	Absolute structure: Flack *x* determined using 2039 quotients [(*I*^+^)-(*I*^-^)]/[(*I*^+^)+(*I*^-^)] (Parsons *et al.*, 2013)
84 restraints	Absolute structure parameter: −0.004 (14)

### Special details

**Table:**

Geometry. All esds (except the esd in the dihedral angle between two l.s. planes) are estimated using the full covariance matrix. The cell esds are taken into account individually in the estimation of esds in distances, angles and torsion angles; correlations between esds in cell parameters are only used when they are defined by crystal symmetry. An approximate (isotropic) treatment of cell esds is used for estimating esds involving l.s. planes.
Refinement. F2 C22 C23 C24 C25 C26 C27 and F2P C22P C23P C24P C25P C26P C27P are disordered over two sites with occupancies 0.83:0.17.The anisotropic ellipsoids of the atoms disordered were elongated, EADP constraint commands in the SHELXL2018 software were used.DELU C22 C23 C24 C25 C26 C27 SIMU 0.04 0.08 1.7 C22 C23 C24 C25 C26 C27 EADP C22 C23 > C27P SIMU 0.04 0.08 1.7 C22P C23P C24P C25P C26P C27P EADP C22P C23P C24P C25P C26P C27P

### Fractional atomic coordinates and isotropic or equivalent isotropic displacement parameters (Å^2^)

**Table:**

	*x*	*y*	*z*	*U*_iso_*/*U*_eq_	Occ. (<1)
Ni1	0.44042 (8)	0.61941 (5)	0.35974 (4)	0.0252 (2)	
P1	0.61916 (13)	0.58432 (8)	0.42665 (6)	0.0283 (3)	
P2	0.58710 (13)	0.71631 (8)	0.31423 (6)	0.0274 (3)	
F1	0.1259 (4)	0.5617 (2)	0.48298 (16)	0.0425 (8)	
O1	0.0663 (4)	0.5316 (2)	0.33306 (17)	0.0326 (7)	
N1	0.3051 (5)	0.5214 (3)	0.3753 (2)	0.0333 (9)	
N2	0.2565 (5)	0.6354 (3)	0.3116 (2)	0.0379 (10)	
C1	0.7924 (6)	0.6365 (3)	0.3979 (3)	0.0340 (11)	
H1A	0.856154	0.649735	0.436486	0.041*	
H1B	0.84555	0.593746	0.368745	0.041*	
C2	0.7616 (5)	0.7237 (3)	0.3599 (3)	0.0329 (10)	
H2A	0.843207	0.735335	0.328437	0.039*	
H2B	0.757134	0.774762	0.391431	0.039*	
C3	0.6012 (6)	0.6231 (4)	0.5139 (2)	0.0354 (11)	
H3	0.693936	0.605893	0.537571	0.042*	
C4	0.5883 (7)	0.7253 (4)	0.5172 (3)	0.0434 (13)	
H4A	0.676569	0.752727	0.49782	0.065*	
H4B	0.578949	0.744174	0.563589	0.065*	
H4C	0.501	0.744741	0.492438	0.065*	
C5	0.4732 (8)	0.5775 (5)	0.5500 (3)	0.0529 (16)	
H5A	0.38004	0.594289	0.528658	0.079*	
H5B	0.471749	0.59715	0.596382	0.079*	
H5C	0.485569	0.511963	0.548109	0.079*	
C6	0.6638 (6)	0.4632 (3)	0.4328 (3)	0.0427 (13)	
H6	0.580353	0.432356	0.455912	0.051*	
C7	0.6776 (7)	0.4229 (4)	0.3633 (4)	0.0515 (15)	
H7A	0.588262	0.436356	0.337786	0.077*	
H7B	0.689946	0.357378	0.366614	0.077*	
H7C	0.763377	0.449	0.340827	0.077*	
C8	0.8040 (9)	0.4454 (5)	0.4720 (5)	0.076 (3)	
H8A	0.88521	0.480585	0.453089	0.114*	
H8B	0.828117	0.381107	0.469959	0.114*	
H8C	0.78893	0.463203	0.518313	0.114*	
C9	0.5258 (6)	0.8346 (3)	0.3051 (2)	0.0335 (11)	
H9	0.441881	0.835256	0.272997	0.04*	
C10	0.4675 (8)	0.8703 (4)	0.3718 (3)	0.0458 (14)	
H10A	0.396569	0.827273	0.390281	0.069*	
H10B	0.418784	0.928579	0.364885	0.069*	
H10C	0.549894	0.87793	0.402721	0.069*	
C11	0.6448 (8)	0.8970 (4)	0.2781 (3)	0.0471 (14)	
H11A	0.730158	0.896204	0.30791	0.071*	
H11B	0.605988	0.958469	0.275035	0.071*	
H11C	0.67496	0.876323	0.234011	0.071*	
C12	0.6424 (6)	0.6818 (3)	0.2294 (3)	0.0341 (11)	
H12	0.712912	0.728128	0.212279	0.041*	
C13	0.5102 (7)	0.6796 (4)	0.1827 (3)	0.0405 (13)	
H13A	0.440577	0.633238	0.197466	0.061*	
H13B	0.543697	0.665672	0.137687	0.061*	
H13C	0.461288	0.738549	0.182946	0.061*	
C14	0.7222 (7)	0.5912 (4)	0.2296 (3)	0.0482 (14)	
H14A	0.811469	0.5956	0.256804	0.072*	
H14B	0.749328	0.574897	0.184152	0.072*	
H14C	0.657041	0.544778	0.248023	0.072*	
C15	0.2713 (6)	0.4580 (3)	0.4236 (3)	0.0306 (10)	
C16	0.3262 (6)	0.3698 (4)	0.4212 (3)	0.0412 (12)	
H16	0.384344	0.351767	0.384349	0.049*	
C17	0.2982 (8)	0.3082 (4)	0.4711 (4)	0.0565 (17)	
H17	0.338353	0.249145	0.468175	0.068*	
C18	0.2127 (7)	0.3314 (5)	0.5249 (4)	0.0519 (16)	
H18	0.194484	0.288984	0.559217	0.062*	
C19	0.1537 (6)	0.4175 (4)	0.5283 (3)	0.0410 (12)	
H19	0.094332	0.434891	0.564956	0.049*	
C20	0.1825 (6)	0.4772 (4)	0.4780 (3)	0.0327 (11)	
C21	0.1934 (6)	0.5609 (3)	0.3393 (2)	0.0292 (10)	
F2	0.1434 (6)	0.5741 (3)	0.1917 (2)	0.0567 (13)	0.832 (7)
C22	0.1725 (9)	0.6924 (4)	0.2689 (4)	0.0422 (7)	0.832 (7)
C23	0.1460 (8)	0.7813 (5)	0.2870 (3)	0.0422 (7)	0.832 (7)
H23	0.184481	0.804054	0.327496	0.051*	0.832 (7)
C24	0.0634 (7)	0.8370 (3)	0.2459 (3)	0.0422 (7)	0.832 (7)
H24	0.045338	0.897811	0.258305	0.051*	0.832 (7)
C25	0.0072 (6)	0.8038 (3)	0.1867 (2)	0.0422 (7)	0.832 (7)
H25	−0.049335	0.841821	0.158588	0.051*	0.832 (7)
C26	0.0336 (6)	0.7148 (3)	0.1685 (2)	0.0422 (7)	0.832 (7)
H26	−0.004865	0.692073	0.128061	0.051*	0.832 (7)
C27	0.1162 (8)	0.6591 (3)	0.2096 (4)	0.0422 (7)	0.832 (7)
F2P	0.181 (2)	0.8178 (13)	0.3304 (10)	0.0422 (7)	0.168 (7)
C22P	0.171 (5)	0.687 (3)	0.2661 (17)	0.0422 (7)	0.168 (7)
C23P	0.129 (4)	0.6397 (17)	0.2097 (19)	0.0422 (7)	0.168 (7)
H23P	0.155659	0.578091	0.204982	0.051*	0.168 (7)
C24P	0.049 (3)	0.6825 (18)	0.1603 (13)	0.0422 (7)	0.168 (7)
H24P	0.02007	0.650124	0.121732	0.051*	0.168 (7)
C25P	0.010 (3)	0.7727 (18)	0.1672 (12)	0.0422 (7)	0.168 (7)
H25P	−0.045066	0.801951	0.133375	0.051*	0.168 (7)
C26P	0.052 (3)	0.8201 (17)	0.2235 (14)	0.0422 (7)	0.168 (7)
H26P	0.025385	0.881747	0.228269	0.051*	0.168 (7)
C27P	0.132 (4)	0.777 (2)	0.2730 (13)	0.0422 (7)	0.168 (7)

### Atomic displacement parameters (Å^2^)

**Table:**

	*U*^11^	*U*^22^	*U*^33^	*U*^12^	*U*^13^	*U*^23^
Ni1	0.0289 (4)	0.0236 (4)	0.0230 (4)	−0.0003 (3)	0.0007 (3)	0.0046 (3)
P1	0.0302 (6)	0.0223 (5)	0.0325 (6)	0.0005 (4)	−0.0035 (5)	0.0024 (5)
P2	0.0372 (6)	0.0214 (5)	0.0237 (6)	−0.0038 (5)	0.0048 (5)	0.0002 (4)
F1	0.0533 (19)	0.0346 (16)	0.0395 (17)	0.0091 (14)	0.0098 (14)	0.0007 (13)
O1	0.0312 (17)	0.0315 (16)	0.0349 (18)	−0.0024 (15)	0.0004 (15)	0.0054 (14)
N1	0.035 (2)	0.036 (2)	0.029 (2)	−0.0063 (18)	0.0019 (17)	0.0022 (18)
N2	0.040 (2)	0.037 (2)	0.037 (2)	−0.0079 (19)	−0.001 (2)	0.009 (2)
C1	0.031 (2)	0.030 (3)	0.040 (3)	−0.004 (2)	0.001 (2)	−0.005 (2)
C2	0.035 (2)	0.033 (2)	0.031 (2)	−0.007 (2)	0.001 (2)	−0.003 (2)
C3	0.044 (3)	0.033 (2)	0.029 (2)	−0.002 (2)	−0.006 (2)	0.004 (2)
C4	0.062 (4)	0.043 (3)	0.025 (3)	0.001 (3)	0.002 (2)	−0.005 (2)
C5	0.064 (4)	0.059 (4)	0.035 (3)	−0.020 (3)	0.002 (3)	0.000 (3)
C6	0.037 (3)	0.024 (2)	0.067 (4)	0.001 (2)	−0.008 (3)	0.007 (3)
C7	0.049 (3)	0.029 (3)	0.077 (4)	0.001 (2)	0.012 (3)	−0.004 (3)
C8	0.061 (4)	0.035 (3)	0.131 (8)	0.002 (3)	−0.043 (5)	0.020 (4)
C9	0.049 (3)	0.025 (2)	0.026 (2)	0.002 (2)	0.001 (2)	0.0000 (19)
C10	0.074 (4)	0.030 (3)	0.033 (3)	0.014 (3)	−0.001 (3)	−0.002 (2)
C11	0.066 (4)	0.029 (3)	0.046 (3)	−0.007 (3)	−0.001 (3)	0.010 (2)
C12	0.040 (3)	0.033 (3)	0.029 (2)	−0.010 (2)	0.009 (2)	−0.007 (2)
C13	0.057 (3)	0.036 (3)	0.029 (3)	−0.009 (2)	0.005 (2)	−0.005 (2)
C14	0.052 (3)	0.047 (3)	0.045 (3)	0.001 (3)	0.008 (3)	−0.021 (3)
C15	0.032 (2)	0.032 (2)	0.028 (2)	−0.005 (2)	0.001 (2)	−0.003 (2)
C16	0.041 (3)	0.036 (3)	0.046 (3)	0.003 (2)	0.006 (2)	−0.007 (3)
C17	0.062 (4)	0.033 (3)	0.075 (5)	0.011 (3)	0.014 (4)	0.014 (3)
C18	0.050 (3)	0.050 (3)	0.056 (4)	0.005 (3)	0.006 (3)	0.024 (3)
C19	0.043 (3)	0.046 (3)	0.034 (3)	0.004 (3)	0.005 (2)	0.006 (2)
C20	0.036 (3)	0.033 (2)	0.030 (3)	0.001 (2)	0.004 (2)	−0.002 (2)
C21	0.039 (3)	0.022 (2)	0.026 (2)	−0.0016 (19)	0.007 (2)	−0.0047 (18)
F2	0.074 (3)	0.048 (2)	0.048 (2)	0.001 (2)	−0.006 (2)	−0.0075 (19)
C22	0.0393 (13)	0.0405 (17)	0.0469 (17)	−0.0004 (12)	0.0024 (12)	0.0121 (15)
C23	0.0393 (13)	0.0405 (17)	0.0469 (17)	−0.0004 (12)	0.0024 (12)	0.0121 (15)
C24	0.0393 (13)	0.0405 (17)	0.0469 (17)	−0.0004 (12)	0.0024 (12)	0.0121 (15)
C25	0.0393 (13)	0.0405 (17)	0.0469 (17)	−0.0004 (12)	0.0024 (12)	0.0121 (15)
C26	0.0393 (13)	0.0405 (17)	0.0469 (17)	−0.0004 (12)	0.0024 (12)	0.0121 (15)
C27	0.0393 (13)	0.0405 (17)	0.0469 (17)	−0.0004 (12)	0.0024 (12)	0.0121 (15)
F2P	0.0393 (13)	0.0405 (17)	0.0469 (17)	−0.0004 (12)	0.0024 (12)	0.0121 (15)
C22P	0.0393 (13)	0.0405 (17)	0.0469 (17)	−0.0004 (12)	0.0024 (12)	0.0121 (15)
C23P	0.0393 (13)	0.0405 (17)	0.0469 (17)	−0.0004 (12)	0.0024 (12)	0.0121 (15)
C24P	0.0393 (13)	0.0405 (17)	0.0469 (17)	−0.0004 (12)	0.0024 (12)	0.0121 (15)
C25P	0.0393 (13)	0.0405 (17)	0.0469 (17)	−0.0004 (12)	0.0024 (12)	0.0121 (15)
C26P	0.0393 (13)	0.0405 (17)	0.0469 (17)	−0.0004 (12)	0.0024 (12)	0.0121 (15)
C27P	0.0393 (13)	0.0405 (17)	0.0469 (17)	−0.0004 (12)	0.0024 (12)	0.0121 (15)

### Geometric parameters (Å, º)

**Table:**

Ni1—N1	1.929 (4)	C10—H10C	0.98
Ni1—N2	1.944 (5)	C11—H11A	0.98
Ni1—P2	2.1631 (14)	C11—H11B	0.98
Ni1—P1	2.1729 (14)	C11—H11C	0.98
Ni1—C21	2.438 (5)	C12—C13	1.526 (8)
P1—C1	1.845 (5)	C12—C14	1.527 (8)
P1—C6	1.846 (5)	C12—H12	1
P1—C3	1.860 (5)	C13—H13A	0.98
P2—C2	1.835 (5)	C13—H13B	0.98
P2—C9	1.850 (5)	C13—H13C	0.98
P2—C12	1.855 (5)	C14—H14A	0.98
F1—C20	1.358 (6)	C14—H14B	0.98
O1—C21	1.238 (6)	C14—H14C	0.98
N1—C21	1.378 (7)	C15—C20	1.391 (7)
N1—C15	1.387 (7)	C15—C16	1.402 (7)
N2—C21	1.364 (6)	C16—C17	1.382 (9)
N2—C22P	1.426 (18)	C16—H16	0.95
N2—C22	1.428 (6)	C17—C18	1.379 (10)
C1—C2	1.529 (7)	C17—H17	0.95
C1—H1A	0.99	C18—C19	1.386 (9)
C1—H1B	0.99	C18—H18	0.95
C2—H2A	0.99	C19—C20	1.373 (8)
C2—H2B	0.99	C19—H19	0.95
C3—C4	1.521 (8)	F2—C27	1.334 (6)
C3—C5	1.528 (8)	C22—C23	1.39
C3—H3	1	C22—C27	1.39
C4—H4A	0.98	C23—C24	1.39
C4—H4B	0.98	C23—H23	0.95
C4—H4C	0.98	C24—C25	1.39
C5—H5A	0.98	C24—H24	0.95
C5—H5B	0.98	C25—C26	1.39
C5—H5C	0.98	C25—H25	0.95
C6—C8	1.520 (9)	C26—C27	1.39
C6—C7	1.531 (9)	C26—H26	0.95
C6—H6	1	F2P—C27P	1.38 (3)
C7—H7A	0.98	C22P—C23P	1.39
C7—H7B	0.98	C22P—C27P	1.39
C7—H7C	0.98	C23P—C24P	1.39
C8—H8A	0.98	C23P—H23P	0.95
C8—H8B	0.98	C24P—C25P	1.39
C8—H8C	0.98	C24P—H24P	0.95
C9—C11	1.522 (8)	C25P—C26P	1.39
C9—C10	1.540 (7)	C25P—H25P	0.95
C9—H9	1	C26P—C27P	1.39
C10—H10A	0.98	C26P—H26P	0.95
C10—H10B	0.98		
			
N1—Ni1—N2	68.13 (19)	C9—C10—H10C	109.5
N1—Ni1—P2	164.07 (14)	H10A—C10—H10C	109.5
N2—Ni1—P2	103.56 (13)	H10B—C10—H10C	109.5
N1—Ni1—P1	101.11 (14)	C9—C11—H11A	109.5
N2—Ni1—P1	168.35 (14)	C9—C11—H11B	109.5
P2—Ni1—P1	87.95 (5)	H11A—C11—H11B	109.5
N1—Ni1—C21	34.35 (17)	C9—C11—H11C	109.5
N2—Ni1—C21	33.96 (17)	H11A—C11—H11C	109.5
P2—Ni1—C21	136.87 (12)	H11B—C11—H11C	109.5
P1—Ni1—C21	134.87 (12)	C13—C12—C14	110.8 (4)
C1—P1—C6	104.0 (3)	C13—C12—P2	111.3 (4)
C1—P1—C3	104.0 (2)	C14—C12—P2	111.6 (4)
C6—P1—C3	104.9 (3)	C13—C12—H12	107.6
C1—P1—Ni1	109.83 (18)	C14—C12—H12	107.6
C6—P1—Ni1	116.0 (2)	P2—C12—H12	107.6
C3—P1—Ni1	116.67 (18)	C12—C13—H13A	109.5
C2—P2—C9	104.6 (2)	C12—C13—H13B	109.5
C2—P2—C12	104.3 (3)	H13A—C13—H13B	109.5
C9—P2—C12	104.6 (2)	C12—C13—H13C	109.5
C2—P2—Ni1	110.91 (17)	H13A—C13—H13C	109.5
C9—P2—Ni1	119.19 (18)	H13B—C13—H13C	109.5
C12—P2—Ni1	112.01 (17)	C12—C14—H14A	109.5
C21—N1—C15	119.7 (4)	C12—C14—H14B	109.5
C21—N1—Ni1	93.5 (3)	H14A—C14—H14B	109.5
C15—N1—Ni1	140.0 (4)	C12—C14—H14C	109.5
C21—N2—C22P	118.2 (19)	H14A—C14—H14C	109.5
C21—N2—C22	120.2 (5)	H14B—C14—H14C	109.5
C21—N2—Ni1	93.3 (3)	N1—C15—C20	122.9 (5)
C22P—N2—Ni1	148.6 (18)	N1—C15—C16	122.0 (5)
C22—N2—Ni1	146.3 (5)	C20—C15—C16	115.1 (5)
C2—C1—P1	110.9 (4)	C17—C16—C15	121.8 (5)
C2—C1—H1A	109.5	C17—C16—H16	119.1
P1—C1—H1A	109.5	C15—C16—H16	119.1
C2—C1—H1B	109.5	C18—C17—C16	120.8 (6)
P1—C1—H1B	109.5	C18—C17—H17	119.6
H1A—C1—H1B	108	C16—C17—H17	119.6
C1—C2—P2	111.0 (3)	C17—C18—C19	119.1 (6)
C1—C2—H2A	109.4	C17—C18—H18	120.4
P2—C2—H2A	109.4	C19—C18—H18	120.4
C1—C2—H2B	109.4	C20—C19—C18	118.9 (5)
P2—C2—H2B	109.4	C20—C19—H19	120.5
H2A—C2—H2B	108	C18—C19—H19	120.5
C4—C3—C5	111.2 (5)	F1—C20—C19	117.9 (5)
C4—C3—P1	110.9 (3)	F1—C20—C15	117.8 (5)
C5—C3—P1	112.4 (4)	C19—C20—C15	124.2 (5)
C4—C3—H3	107.4	O1—C21—N2	129.3 (5)
C5—C3—H3	107.4	O1—C21—N1	126.1 (4)
P1—C3—H3	107.4	N2—C21—N1	104.6 (4)
C3—C4—H4A	109.5	O1—C21—Ni1	176.0 (4)
C3—C4—H4B	109.5	N2—C21—Ni1	52.8 (3)
H4A—C4—H4B	109.5	N1—C21—Ni1	52.2 (2)
C3—C4—H4C	109.5	C23—C22—C27	120
H4A—C4—H4C	109.5	C23—C22—N2	119.7 (5)
H4B—C4—H4C	109.5	C27—C22—N2	120.3 (5)
C3—C5—H5A	109.5	C24—C23—C22	120
C3—C5—H5B	109.5	C24—C23—H23	120
H5A—C5—H5B	109.5	C22—C23—H23	120
C3—C5—H5C	109.5	C25—C24—C23	120
H5A—C5—H5C	109.5	C25—C24—H24	120
H5B—C5—H5C	109.5	C23—C24—H24	120
C8—C6—C7	110.0 (6)	C24—C25—C26	120
C8—C6—P1	112.8 (4)	C24—C25—H25	120
C7—C6—P1	109.7 (4)	C26—C25—H25	120
C8—C6—H6	108.1	C27—C26—C25	120
C7—C6—H6	108.1	C27—C26—H26	120
P1—C6—H6	108.1	C25—C26—H26	120
C6—C7—H7A	109.5	F2—C27—C26	119.9 (5)
C6—C7—H7B	109.5	F2—C27—C22	120.1 (5)
H7A—C7—H7B	109.5	C26—C27—C22	120
C6—C7—H7C	109.5	C23P—C22P—C27P	120
H7A—C7—H7C	109.5	C23P—C22P—N2	114 (3)
H7B—C7—H7C	109.5	C27P—C22P—N2	126 (3)
C6—C8—H8A	109.5	C24P—C23P—C22P	120
C6—C8—H8B	109.5	C24P—C23P—H23P	120
H8A—C8—H8B	109.5	C22P—C23P—H23P	120
C6—C8—H8C	109.5	C25P—C24P—C23P	120
H8A—C8—H8C	109.5	C25P—C24P—H24P	120
H8B—C8—H8C	109.5	C23P—C24P—H24P	120
C11—C9—C10	110.3 (5)	C24P—C25P—C26P	120
C11—C9—P2	113.5 (4)	C24P—C25P—H25P	120
C10—C9—P2	110.1 (3)	C26P—C25P—H25P	120
C11—C9—H9	107.6	C27P—C26P—C25P	120
C10—C9—H9	107.6	C27P—C26P—H26P	120
P2—C9—H9	107.6	C25P—C26P—H26P	120
C9—C10—H10A	109.5	F2P—C27P—C26P	125 (2)
C9—C10—H10B	109.5	F2P—C27P—C22P	115 (2)
H10A—C10—H10B	109.5	C26P—C27P—C22P	120
			
C6—P1—C1—C2	153.4 (4)	C16—C15—C20—C19	3.0 (8)
C3—P1—C1—C2	−97.0 (4)	C22P—N2—C21—O1	−4 (2)
Ni1—P1—C1—C2	28.6 (4)	C22—N2—C21—O1	0.3 (9)
P1—C1—C2—P2	−33.2 (5)	Ni1—N2—C21—O1	175.6 (5)
C9—P2—C2—C1	154.7 (4)	C22P—N2—C21—N1	175 (2)
C12—P2—C2—C1	−95.8 (4)	C22—N2—C21—N1	178.4 (5)
Ni1—P2—C2—C1	24.9 (4)	Ni1—N2—C21—N1	−6.2 (4)
C1—P1—C3—C4	62.0 (5)	C22P—N2—C21—Ni1	−179 (2)
C6—P1—C3—C4	171.0 (4)	C22—N2—C21—Ni1	−175.4 (6)
Ni1—P1—C3—C4	−59.1 (4)	C15—N1—C21—O1	−18.8 (7)
C1—P1—C3—C5	−172.8 (4)	Ni1—N1—C21—O1	−175.5 (4)
C6—P1—C3—C5	−63.9 (5)	C15—N1—C21—N2	163.0 (4)
Ni1—P1—C3—C5	66.0 (5)	Ni1—N1—C21—N2	6.3 (4)
C1—P1—C6—C8	50.5 (6)	C15—N1—C21—Ni1	156.7 (5)
C3—P1—C6—C8	−58.5 (6)	C21—N2—C22—C23	117.0 (5)
Ni1—P1—C6—C8	171.2 (5)	Ni1—N2—C22—C23	−54.7 (10)
C1—P1—C6—C7	−72.4 (4)	C21—N2—C22—C27	−62.5 (7)
C3—P1—C6—C7	178.6 (4)	Ni1—N2—C22—C27	125.8 (7)
Ni1—P1—C6—C7	48.3 (4)	C27—C22—C23—C24	0
C2—P2—C9—C11	50.1 (4)	N2—C22—C23—C24	−179.5 (7)
C12—P2—C9—C11	−59.2 (4)	C22—C23—C24—C25	0
Ni1—P2—C9—C11	174.8 (3)	C23—C24—C25—C26	0
C2—P2—C9—C10	−74.1 (4)	C24—C25—C26—C27	0
C12—P2—C9—C10	176.6 (4)	C25—C26—C27—F2	−179.0 (6)
Ni1—P2—C9—C10	50.6 (5)	C25—C26—C27—C22	0
C2—P2—C12—C13	−175.5 (4)	C23—C22—C27—F2	179.0 (6)
C9—P2—C12—C13	−65.9 (4)	N2—C22—C27—F2	−1.5 (6)
Ni1—P2—C12—C13	64.5 (4)	C23—C22—C27—C26	0
C2—P2—C12—C14	60.2 (4)	N2—C22—C27—C26	179.5 (7)
C9—P2—C12—C14	169.7 (4)	C21—N2—C22P—C23P	−62 (2)
Ni1—P2—C12—C14	−59.8 (4)	Ni1—N2—C22P—C23P	120 (3)
C21—N1—C15—C20	−60.0 (7)	C21—N2—C22P—C27P	120 (2)
Ni1—N1—C15—C20	82.2 (7)	Ni1—N2—C22P—C27P	−58 (5)
C21—N1—C15—C16	120.9 (6)	C27P—C22P—C23P—C24P	0
Ni1—N1—C15—C16	−97.0 (7)	N2—C22P—C23P—C24P	−178 (3)
N1—C15—C16—C17	176.7 (6)	C22P—C23P—C24P—C25P	0
C20—C15—C16—C17	−2.5 (9)	C23P—C24P—C25P—C26P	0
C15—C16—C17—C18	0.9 (11)	C24P—C25P—C26P—C27P	0
C16—C17—C18—C19	0.5 (11)	C25P—C26P—C27P—F2P	179 (3)
C17—C18—C19—C20	−0.1 (10)	C25P—C26P—C27P—C22P	0
C18—C19—C20—F1	−178.8 (5)	C23P—C22P—C27P—F2P	−179 (3)
C18—C19—C20—C15	−1.8 (9)	N2—C22P—C27P—F2P	−1 (3)
N1—C15—C20—F1	0.8 (8)	C23P—C22P—C27P—C26P	0
C16—C15—C20—F1	180.0 (5)	N2—C22P—C27P—C26P	178 (4)
N1—C15—C20—C19	−176.2 (5)		

### Hydrogen-bond geometry (Å, º)

**Table:**

*D*—H···*A*	*D*—H	H···*A*	*D*···*A*	*D*—H···*A*
C1—H1*B*···O1^i^	0.99	2.32	3.210 (6)	149
C10—H10*C*···F1^ii^	0.98	2.57	3.416 (7)	145
C5—H5*A*···F1	0.98	2.53	3.435 (8)	154
C8—H8*A*···F1^i^	0.98	2.56	3.398 (8)	143
C4—H4*B*···F2*P*^ii^	0.98	2.5	3.25 (2)	133
C10—H10*A*···F2*P*	0.98	2.31	2.84 (2)	114

Symmetry codes: (i) *x*+1, *y*, *z*; (ii) *x*+1/2, −*y*+3/2, −*z*+1.
